# Online decision aids for contraceptive choices in women with chronic conditions: A systematic review

**DOI:** 10.1002/ijgo.70555

**Published:** 2025-09-27

**Authors:** Begashaw Melaku Gebresillassie, Nicole Doley, Melissa L. Harris

**Affiliations:** ^1^ School of Medicine and Public Health The University of Newcastle, University Drive Callaghan New South Wales Australia; ^2^ Centre for Women's Health Research The University of Newcastle, University Drive Callaghan New South Wales Australia; ^3^ Hunter Medical Research Institute Kookaburra Circuit New Lambton New South Wales Australia

**Keywords:** chronic conditions, contraceptive decision aids, web‐based interventions

## Abstract

**Background:**

Women with chronic conditions face increased risks of unintended pregnancy and adverse maternal and perinatal outcomes. Tailored, online contraceptive decision aids might improve informed decision‐making and support reproductive health by promoting patient‐centered care in this population.

**Objectives:**

This study systematically reviews the characteristics and effectiveness of online decision aids designed to support contraceptive and reproductive health choices in women with chronic conditions.

**Search Strategy:**

We searched Medline, Embase, CINAHL, Maternity and Infant Care Database, and Emcare up until November 30, 2024, using terms related to contraception, decision aids, chronic conditions, and women's health. We also searched thesis repositories and reference lists of relevant reviews.

**Selection Criteria:**

Included studies evaluated web‐based decision aids with a contraceptive choice component, targeted women of reproductive age (15–50 years) with chronic conditions, and assessed outcomes related to feasibility, user experience, knowledge, communication, or contraceptive uptake and behavior change.

**Data Collection and Analysis:**

Two reviewers independently extracted data on study characteristics, intervention features, and outcomes and assessed methodological quality using the Mixed Methods Appraisal Tool (MMAT). A narrative synthesis was conducted due to heterogeneity in study designs and outcomes. The GRADE approach was not applied to assess the quality of the included studies due to variability in study designs and outcomes. The protocol was registered with PROSPERO (CRD42023473313).

**Main Results:**

Ten studies evaluating nine distinct decision aids met the inclusion criteria. Eight focused primarily on contraceptive choices, while two addressed broader reproductive health planning. Most studies were conducted in the USA (*n* = 8) and employed various designs: randomized controlled trials (*n* = 4), mixed‐methods studies (*n* = 2), and descriptive studies (*n* = 3). The decision aids targeted various chronic conditions, including hypertension, diabetes, cancer, rheumatic diseases, cystic fibrosis, and sickle cell disease. The aids incorporated interactive features, personalized recommendations, and multimedia formats, with high user satisfaction and usability ratings reported. Some decision aids demonstrated improvements in contraceptive use, reproductive health knowledge, and communication. However, the effect on long‐term contraceptive behaviors was mixed, with three studies showing positive but not statistically significant changes and one study reporting a negative correlation.

**Conclusions:**

Online decision aids tailored for women with chronic conditions showed promise in improving user satisfaction, reproductive health knowledge, and patient–provider communication. However, their impact on long‐term contraceptive behaviors remains inconclusive. Future studies should employ rigorous designs, validated outcome measures, and larger, more diverse populations to further evaluate the effectiveness of these aids and optimize their impact on contraceptive decision‐making in this population.

## 1 INTRODUCTION

The prevalence of chronic conditions, such as asthma, hyperlipidaemia, cardiac conditions (including hypertensive heart disease, heart failure, coronary artery disease), diabetes, obesity, and psychiatric disorders, has increased among reproductive‐age women over the past decade.^1^, ^2^ Up to 45% of women seen in primary care have chronic conditions.^2^ These women face higher risks of pregnancy complications and mortality compared to those without such conditions,^3^, ^4^, ^5^, ^6^, ^7^ underscoring the importance of planning pregnancies during periods of better health and adjusting medications as needed. At the same time, disparities in contraceptive use remain evident, with some women relying on less effective methods, which might increase the likelihood of unintended pregnancies.^8^, ^9^, ^10^, ^11^, ^12^

Unintended pregnancies for women with chronic conditions are associated with serious maternal and perinatal outcomes, such as congenital abnormalities, early pregnancy loss, and stillbirth.^11^ Further, women with chronic conditions, such as cardiac and autoimmune diseases, often take medications that is contraindicated to pregnancy,^13^ emphasizing the importance of contraceptive counseling that is tailored to their specific health profiles and includes information on potential drug interactions and pregnancy‐compatible medications. Despite its importance, contraceptive counseling for women with chronic conditions remains inadequate.

Women with chronic conditions need tailored contraceptive counseling and advice.^8^, ^9^, ^11^, ^14^ However, many of these women have reported that the information provided by healthcare professionals is often substandard, with healthcare providers not fully addressing their unique needs and complexities.^15^, ^16^ While provider recommendations significantly influence contraceptive choices, generic contraceptive information alone is inadequate.^17^, ^18^ Decision aids, particularly those delivered online, can bridge this gap by facilitating effective communication between women and healthcare professionals.^19^, ^20^ These tools support patient‐centered communication by providing personalized, interactive information, such as visual aids and options to compare contraceptive methods, which is crucial for those facing complex health decisions.^20^, ^21^ Online decision aids offer unique advantages for women with chronic conditions, including increased accessibility, privacy, and the ability to tailor information and recommendations based on individual health profiles and medication regimens.^20^, ^21^ Despite their benefits, existing decision aids are often designed for the general population and lack the specificity needed for women with chronic conditions, who require comprehensive and customized support to make informed choices about contraception and reproductive health.

With the advancements of technology in health care, decision aids have evolved to utilize web‐based platforms and interactive applications.^22^ This progression has made information and decision‐making more accessible to patients. Available evidence on decision aids in contraceptive services has focused on various modalities, including both paper‐based and technology‐based aids, targeting the general population.^20^, ^23^ However, there is limited evidence on online contraceptive decision aids specifically designed for women with chronic conditions. Given that women with chronic conditions face unique challenges when making contraceptive decisions, the use of these aids can be particularly valuable in facilitating informed choices.^3^, ^11^, ^13^, ^24^

It is crucial to synthesize evidence on decision aids tailored for this population, as this will provide them with the necessary support, information, and tools to make informed contraception and healthcare decisions, ultimately improving their overall well‐being. In this rapidly evolving area,^25^, ^26^ synthesizing the current evidence is essential to optimize the design and facilitate the implementation of online contraceptive decision aids for women with chronic conditions, ensuring improved access to comprehensive contraception and health information, as well as decision support. Therefore, this review aimed to systematically review the available online decision aids for women with chronic conditions, assessing their characteristics and evaluating their effectiveness in supporting women with chronic conditions, specifically in areas of contraceptive uptake, pregnancy planning, and information about chronic conditions.

## 2 METHODS

This systematic review was conducted and reported in accordance with the Preferred Reporting Items for Systematic reviews and Meta‐Analysis Protocols (PRISMA‐P).^27^ The protocol for the review was registered in the PROSPERO International prospective register of systematic reviews (Registration Number: CRD42023473313).

### 2.1 Information sources, search strategy, and study selection

We conducted a systematic search across multiple databases, including Medline, Embase, CINAHL, Maternity and Infant Care Database, and Emcare, to ensure a thorough exploration of the relevant literature. To capture a broader range of studies, we extended our search to include additional sources, such as thesis repositories and Google Scholar. Further, an additional search was performed by examining the reference lists of related systematic reviews. This process aimed to identify any additional eligible studies that might not have been captured through the primary database searches.

The comprehensive search strategy, developed by BMG and MLH in collaboration with the University of Newcastle librarian, combined controlled vocabulary and free‐text terms covering three domains: (i) decision support and counseling (e.g., decision‐making, decision aid, shared decision, and patient education); (ii) online or technology‐based delivery (e.g., internet, web‐based, and telemedicine); and (iii) contraception and family planning (e.g., contraceptive methods, family planning services, and fertility regulation). The search strategy was initially tailored for Medline and adapted for other databases, ensuring consistency and relevance across different search engines. Appropriate limits were applied, and studies conducted up until November 30, 2024, were retrieved. The full search strategy is provided in Table S1. Studies from all comprehensive searches of electronic databases and other sources were exported as EndNote files (including titles and abstracts) and then imported into EndNote as a single library. Duplicate studies resulting from the searches were verified using EndNote's duplicate identification feature and then removed. The remaining studies were imported into the Covidence platform for further screening.^28^ Two reviewers (BMG and ND) independently screened all retrieved studies by titles and abstracts. Abstracts and full texts of the selected studies were then assessed using the predefined eligibility criteria. In case of any disagreements, a third reviewer, MLH, was consulted to make the final decision.

### 2.2 Eligibility criteria

#### Population

Studies that included adolescents or women of reproductive age (15–50 years) who were sexually active or at risk of unintended pregnancies (defined as not being currently pregnant, not trying to become pregnant, or having a partner who could have children) and were considering or seeking information about contraception methods were included. Only human studies written in English were considered.

#### Intervention

The intervention comprised online or web‐based contraceptive decision aids, either standalone or combined with other formats (e.g., mobile applications with an online component), designed to include at least one component, or included content specifically related to chronic conditions. Any chronic condition reported in the studies was considered for inclusion. These aids could involve screening and/or counseling on various contraceptive methods, evaluating contraceptive needs, assessing benefits and risks, providing recommendations based on individual characteristics and medical conditions, delivering education, and assessing patient preferences. While the primary focus was on decision aids specifically designed for contraceptive choices in women with chronic conditions, we also included aids that address broader reproductive health planning for women with specific chronic conditions if they incorporated a substantial component related to contraceptive options. Conversely, interventions that were not specific to contraception did not include components related to chronic conditions or were not available online (e.g., those exclusively used by clinical staff, paper‐based, or telephone interventions) were excluded.

#### Study type and design

The review included original research articles utilizing quantitative, qualitative, or mixed‐method research designs to maximize the inclusion of relevant studies on online contraceptive decision aids. Eligible studies specifically focused on the development, implementation, or evaluation of these aids. In contrast, reviews, protocols, editorials, and commentaries were excluded. Additionally, studies with missing abstracts and full texts were excluded if the authors were contacted and did not respond to the request.

#### Outcome measures

The review considered a broad range of outcomes related to the impact or effectiveness of the decision aids, including the uptake of contraceptive methods, pregnancy planning, and patient experiences. Additionally, studies reporting negative outcomes or non‐significant findings in these areas were included to provide a comprehensive understanding of the effectiveness of the decision aids.

#### Data extraction

Data extraction was independently conducted by two authors (BMG and ND), thereby extracting all relevant information from the included articles into a standardized Excel spreadsheet. Data extracted included study and participant characteristics, context, study design, methodology, intervention/decision aid features and content, and outcome measures, following pre‐defined criteria outlined in the Joanna Briggs Institute guideline.^29^ Subsequently, the two authors cross‐checked their extractions through a collaborative process, discussing any discrepancies and reaching a consensus.

#### Assessment of risk of bias

The quality of the included studies was assessed using the Mixed Methods Appraisal Tool (MMAT).^30^ The MMAT provides a standardized approach for evaluating diverse study designs. Each study was categorized according to its design and evaluated against the relevant MMAT criteria. Ratings of “Yes,” “No,” or “Can't tell” were assigned to indicate whether each criterion was met.

#### Data synthesis

Data for this review were synthesized narratively to comprehensively address the research questions, considering the diverse methodological approaches and outcomes assessed across the included studies. Findings have been descriptively presented and discussed, elaborating on the characteristics of the interventions/online decision aids, and outcomes. Due to the heterogeneity in the methodological approaches used and the diversity in outcomes, a meta‐analysis was not conducted.

## 3 RESULTS

### 3.1 Search results and study selection

**FIGURE 1:**
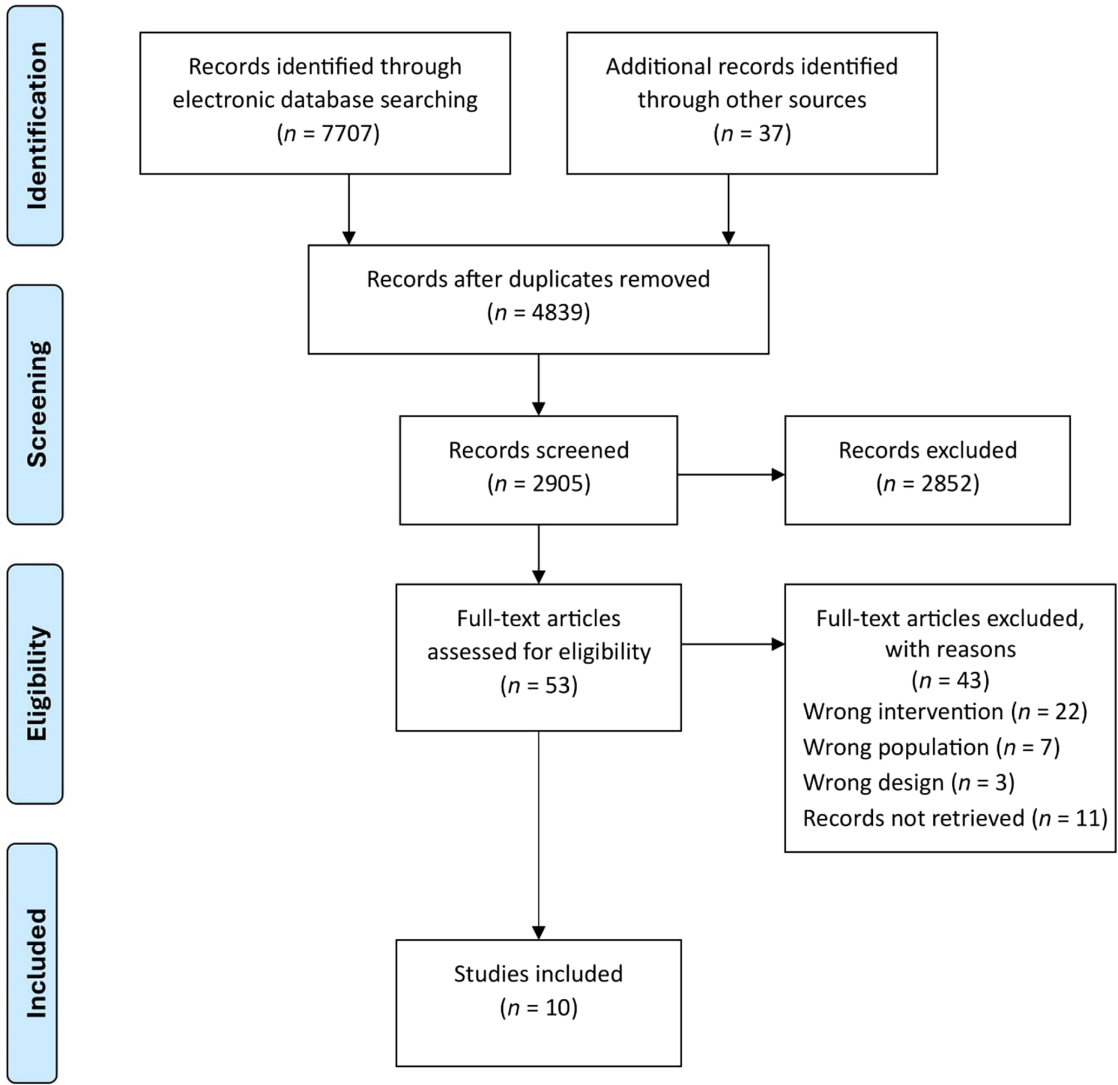
PRISMA flow diagram illustrating the study selection.

A comprehensive search across electronic databases yielded a total of 7707 studies, while an additional 37 studies were identified through other sources. After the removal of 4839 duplicates, 2905 studies underwent initial screening based on titles and abstracts. Subsequently, 2852 studies were excluded during this initial screening phase. A total of 53 studies progressed to a more detailed full text assessment against the inclusion and exclusion criteria. Following a detailed evaluation, 43 studies were excluded, with the main reasons being online decision aid not being focused on contraceptive counseling (*n* = 22), population (*n* = 7), design (*n* = 3), and unretrievable records (*n* = 11). As a result, a final set of 10 studies met the criteria for inclusion in this systematic review, with sample sizes ranging from nine to 9775. The flow diagram in Figure 1 depicts the entire review process.

### 3.2 Study characteristics

In this review, we analyzed 10 studies, with the majority (*n* = 8, 80%) conducted in the USA.^14^, ^31^, ^32^, ^33^, ^34^, ^35^, ^36^, ^37^ The remaining two studies^38^, ^39^ were conducted in multiple countries, with one involving participants mainly from the USA and Canada.^38^ These studies primarily focused on developing and evaluating online contraception decision aids with components related to chronic conditions and tailored for women with chronic conditions such as hypertension, anxiety, depression, diabetes, cancer, rheumatic diseases, cystic fibrosis, and sickle cell disease or trait. Specifically, nine studies assessed the effectiveness, usability, feasibility, and integration of these aids into health care,^14^, ^31^, ^32^, ^33^, ^35^, ^36^, ^37^, ^38^, ^39^ while the remaining study exclusively described the detailed development of the decision aid.^34^ The included studies employed various study designs, including four randomized controlled trials (RCTs), three quantitative descriptive studies, two mixed‐methods studies, and one pre‐post intervention study (Table 1). The majority (*n* = 5, 50%) of these studies recruited participants from clinical settings, such as clinics, hospitals, and medical/health centers.^14^, ^32^, ^35^, ^36^, ^37^ Participants' ages ranged from 15 to 50 years, with the majority focusing on young adults. The sample size of the included studies varied widely, ranging from as few as nine in a pilot feasibility study^34^ to 9775 in extensive evaluations^38^ (Table 1).

**TABLE 1:** Characteristics of studies on online decision aids for women with chronic conditions.

First author, year	Country	Study design	Setting	Chronic condition(s)	Age in years (range or mean ± SD)	Sample size
Redman, 2023^31^	USA	Mixed methods	College	Hypertension, mental health disorders, coagulation disorder	18–24	150
Himes, 2017^32^	USA	Randomized controlled trial	Hospital	Hypertension, diabetes	18–50	59
Benedict, 2022^33^	USA	Mixed methods	Online	Cancer	30.78 ± 4.51	10
Talabi, 2022^34^	USA	Qualitative	Online	Rheumatic diseases	–	9
Kazmerski, 2023^35^	USA	Single arm pre‐post intervention	Clinics	Cystic fibrosis	30.34 ± 5.70	39
Gallo, 2016^14^	USA	Randomized controlled trial	Clinics, community	Sickle cell disease or trait	18–35	234
Batra, 2018^36^	USA	Randomized controlled trial	Medical center	Not restricted^a^	18–45	292
Garbers, 2012^37^	USA	Randomized controlled trial	Clinics	Not restricted^a^	27.7	2231
Nguyen, 2010^38^	Not specified^b^	Descriptive	Online	Not restricted^a^	<35	9775
Nguygen, 2011^39^	Not specified^b^	Descriptive	Online	Not restricted^a^	–	Adolescents (*n* = 3178) adults (*n* = 4206)

Abbreviation: SD, standard deviation.

^a^ Participants with any diagnosed chronic condition were eligible. The decision aid considered the identified chronic conditions.

^b^ Participants were recruited online and not restricted to specific countries.

### 3.3 Features and characteristics of the online decision aids

Table 2 summarizes the features and content of nine online decision aids designed to support women with chronic conditions in making informed decisions about contraception and reproductive health. Seven of these aids focus on contraceptive choices, either as their primary purpose or as a major component within a broader reproductive health framework.^31^, ^32^, ^34^, ^35^, ^36^, ^37^, ^38^, ^39^ These tools offer personalized guidance on contraceptive methods, taking into account the specific health considerations of women with chronic conditions. The remaining two aids, “CHOICES”^14^ and “Roadmap to Parenthood,”^33^ primarily address family planning and pregnancy planning for women with sickle cell disease and cancer, respectively. However, they also incorporated information on contraceptive options and discussed the impact of these conditions on pregnancy planning.

**TABLE 2:** Features and content of online decision aids for women with chronic conditions.

Author, year	Decision aid	Chronic condition(s) covered	Content related to chronic condition(s)	General features
Redman, 2023^31^	My Contraceptive Choice	Hypertension, mental health disorders, coagulation disorder	Personalized decision support based on chronic conditions (blood clotting, mental health, hypertension), medication side effects, and weight management	Contraceptive options Decision supports based on: Preferences: Cost, efficacy, menstrual control, side effects User history: Previous methods, experiences Other Factors: Insertion comfort, hormone preference, maintenance frequency
Himes, 2017^32^	Healthy Beyond Pregnancy	Hypertension, diabetes	Videos on gestational diabetes, hypertension, and preterm birth Algorithm‐directed video selection based on user ratings	Survey assessing postpartum concerns Scheduling of postpartum visits via Healthy Beyond Pregnancy Nudging text reminders for participants Cash incentives for postpartum visit returnees
Benedict, 2022^33^	Roadmap to Parenthood	Cancer	Information on fertility and family‐building options Choices include natural contraception, IUI, IVF, surrogacy with a gestational carrier, adoption, and family preservation post‐treatment Real stories from women sharing their experiences	–
Talabi, 2022^34^	MyVoice:Rheum	Rheumatic diseases	Tailored information on pregnancy and parenting, specifically disease‐specific details on pregnancy, breastfeeding, contraception, and family planning considering their health conditions	–
Kazmerski, 2023^35^	MyVoice:CF	Cystic fibrosis	Tailored information on pregnancy and parenting, specifically disease‐specific details on pregnancy, breastfeeding, contraception, and family planning considering their health conditions	–
Gallo, 2016^14^	CHOICES	Sickle cell disease or trait	Concrete experience: Video of men discussing SCD Reflective observation: Participants answer post‐video questions Abstract conceptualization: Information on genetic risk, reproductive/contraception options for SCD/SCT Active experimentation: Couples' decision videos; personalized parenting plan generated and confirmed by participant	–
Batra, 2018^36^	MyFamilyPlan	Not restricted^a^	Information on chronic medical conditions and corresponding prescribed medications	Preconception health education and self‐assessment items covering nutrition, immunizations, substance use, family/genetic history, environmental exposures, medications, obstetric history, and chronic medical conditions, as outlined in national preconception health guidelines
Garbers, 2012^37^	Computer‐Based Contraceptive Assessment Module	Not restricted^a^	Algorithm accounts for user's medical history including chronic health conditions	Self‐administered computer module Customizable algorithm considering patient preferences Accounts for medical, obstetric, gynecologic, and contraceptive history Evaluates sexual health risk factors
Nguyen, 2010^38^	Choosing Wisely	Not restricted^a^	Records medical/chronic conditions Logs concerns related to chronic conditions Tracks issues like weight gain, headaches, cancer, acne, bone density	Program asks a series of user questions Generates ideal contraceptive choices Provides list of ill‐advised methods Includes personalized explanations for each selection
Nguygen, 2011^39^	Choosing Wisely	Not restricted^a^	Records medical/chronic conditions Logs concerns related to chronic conditions Tracks issues like weight gain, headaches, cancer, acne, bone density	Program asks a series of user questions Generates ideal contraceptive choices Provides list of ill‐advised methods Includes personalized explanations for each selection

Abbreviations: CF, cystic fibrosis; IUI, intrauterine insemination; IVF, in vitro fertilization; rheum, rheumatic disease; SCD, sickle cell disease; SCT, sickle cell trait.

^a^ Participants with any diagnosed chronic condition were eligible. The decision aid considered the identified chronic conditions.

The reviewed online decision aids shared several key features designed to improve user engagement and support informed decision‐making. One of the commonly shared characteristics was the incorporation of interactive and tailored content.^31^, ^37^, ^38^, ^39^ Many decision aids employed interactive elements, such as quizzes or questionnaires, to generate personalized recommendations. For instance, “My Contraceptive Choice,” which takes into account the presence of chronic conditions such as hypertension, mental disorders, and coagulation disorders, utilized a brief quiz to provide customized birth control recommendations,^31^ while “Choosing Wisely,” which takes into account the presence of various chronic conditions, generated lists of indicated, suitable, and contraindicated contraceptive methods based on a self‐administered questionnaire.^38^, ^39^ Similarly, the “Computer‐Based Contraceptive Assessment Module” offered tailored output based on an algorithm that accounted for user‐specific preferences and medical history.^37^

Some decision aids leveraged multimedia formats to improve engagement and knowledge transfer, recognizing the diverse learning styles and preferences among users. Notably, the “CHOICES” program used a web‐based multimedia approach to educate individuals with sickle cell disease or trait about contraception and reproductive health decisions.^14^ In addition, some decision aids extended their functionality beyond information provision by including tools for decision support and planning. “Roadmap to Parenthood,” for example, incorporated resources for values clarification, family‐building stories, and action planning to assist individuals facing family‐building decisions after cancer.^33^ “MyVoice:Rheum” decision aid designed for women with rheumatic diseases promoted patient autonomy by presenting options for family formation alongside a digital notepad for users to record thoughts and questions for their healthcare providers.^34^ Further, accessibility was also prioritized in the design of these interventions. The “Computer‐Based Contraceptive Assessment Module” employed audio‐computer‐assisted self‐interviewing and touchscreen technologies to accommodate users with varying literacy levels^37^ (Table 2).

### 3.4 Outcome evaluation and effectiveness

To understand the impact of decision aids, results were organized by outcomes evaluated including (i) feasibility, usability, and user satisfaction; (ii) reproductive health knowledge, pregnancy planning, and communication; and (iii) contraceptive decision‐making, uptake, and behaviors (Table 3).

**TABLE 3:** Summary of key findings on online decision aids for women with chronic conditions.

First author, year	Decision aid	Key findings	Limitations
Redman, 2023^31^	My Contraceptive Choice	High user satisfaction; 75% expressed positive experiences with learning about birth control methods A top concern is weight gain as a decision factor Simulated tests showed that the tool had an accuracy rate of 72% in matching user preferences and needs	College participant recruitment might not reflect the general public; lab‐based simulated tests limit real‐world applicability
Himes, 2017^32^	Healthy Beyond Pregnancy	High usability, median PSSUQ score 65, IQR: 61–7; positive user feedback led to program enhancements 100% web‐based program completion rate, and willingness to receive text messages 85% postpartum visit return in intervention arm vs. 53% control	Small sample size; more nulliparous women in intervention arm might skew postpartum visit adherence
Benedict, 2022^33^	Roadmap to Parenthood	Improved perceived ease of use from “Acceptable” (score 68) to “Excellent” (89.4) after design updates User evaluation scores for perceived usefulness, ease of use, and intent to reuse increased from 5.6 to 6.25 (range 1–7) Enhanced confidence in managing family‐building; encouraged active role in fertility decisions 10% experienced negative emotions regarding infertility risks and challenges	Small sample size; participant demographics skewed towards white, and highly educated
Talabi, 2022^34^	MyVoice:Rheum	Beta testing with 9 users showed unanimous agreement that the tool offered salient, trustworthy, and clear information on family planning and rheumatic diseases	Not reported
Kazmerski, 2023^35^	MyVoice:CF	Acceptability at 4.47 ± 0.50 and appropriateness at 4.63 ± 0.48 out of 5, indicating high approval and suitability Usability scored an “A” excellent with 82.69 on the System Usability Scale Reproductive health communication self‐efficacy improved significantly (3.62 to 4.03, *P* < 0.001) Improved discussion on reproductive goals with CF team from 33% to 56% post‐intervention (*P* = 0.046)	Not reported
Gallo, 2016^14^	CHOICES	Intervention group showed greater knowledge improvement than control (*P* = 0.004), no change in intention (*P* = 0.18) or behavior (*P* = 0.69) More participants in the intervention group had more risk‐reducing partner choices (*P* = 0.04)	Primarily African descent participants; applicability to other ethnic groups uncertain; cognitive impairment impact among SCD patients not assessed
Batra, 2018^36^	MyFamilyPlan	Linked to increased discussion of reproductive health (OR 1.97; 95% CI: 1.22–3.19) No significant changes in folate supplementation, additional appointments, or self‐efficacy Negative association found between exposure and birth control changes 73.3% of the intervention arm liked the online format	Self‐reported outcomes vulnerable to bias; participant demographics skewed towards white, healthy, privately insured; low enrolment acceptance rate (<10%) raises feasibility concerns
Garbers, 2012^37^	Computer‐Based Contraceptive Assessment Module	74% in the Intervention + Tailored group chose an effective method (AOR = 1.40; 95% CI: 1.11–1.75) 76% in the Intervention + Generic group chose an effective method (AOR = 1.58; 95% CI: 1.24–2.02) 22% in the Intervention + Tailored group and 24% in the Intervention + Generic group chose a top‐tier effective method (mostly intrauterine devices, one vasectomy) versus 15% in the Control group	Unequal randomization across arms reduced statistical power; unmeasured confounding due to controls being recruited later; limited generalizability to non‐Latina populations
Nguyen, 2010^38^	Choosing Wisely	In the first 13 months: 72.7% view pregnancy as devastating; 66.5% confident in remembering daily pill 48% reported menstrual issues; 33.4% of these unsure about daily pill adherence Top lifestyle plan is uncertainty about having children (48%), then planning within 5 years 75.3%reported at least one concern about contraceptive options, with weight gain (65.3% of concerns) being the most common	Program did not distinguish between new and repeat users; lack of user data on exact age and location
Nguygen, 2011^39^	Choosing Wisely	In six‐month period: Adolescents find pregnancy devastating compared to adults (*P* < 0.01) 29% of adolescents with problem menses (crampy, heavy and/or irregular) unable to take a pill daily; 35% of adults with similar issues felt the same Both adolescents (73%) and adults (71%) willing to use contraceptives requiring intercourse interruption	Self‐reported data might introduce biases; potential limitations in tracking user sessions and identifying new vs. repeat users; user base might not reflect the broader population of women with chronic conditions; questionnaire lacks external validation

Abbreviation: AOR, adjusted odds ration; CF, cystic fibrosis; CI, confidence interval; IQR, interquartile range; PSSUQ, post‐study system usability questionnaire; rheum, rheumatic disease.

### 3.5 Feasibility, usability, and user satisfaction

Some studies demonstrated the feasibility, high usability, and user satisfaction of online decision aids for women with chronic conditions. “MyVoice:CF,” a tool designed for women with cystic fibrosis, received high ratings for acceptability (mean = 4.47 out of 5) and appropriateness (mean = 4.63 out of 5).^35^ Its System Usability Scale (SUS) score was 82.69 out of 100, indicating “excellent” usability.^35^ Similarly, “Healthy Beyond Pregnancy,” a web‐based intervention aimed at improving postpartum visit attendance, achieved a median Post‐Study System Usability Questionnaire (PSSUQ) score of 6.5 out of 7, also indicating high usability.^32^ The “Roadmap to Parenthood,” a decision aid for family building after cancer, showed improved usability following design modifications, informed by user feedback. Its SUS scores increased from an average of 68 (“acceptable”) to 89.4 (“excellent”) after these changes, highlighting the importance of iterative, user‐centered design.^33^ User evaluation based on WebQual scores for this decision aid also improved, rising from 5.6 to 6.25 out of 7.^33^ Further, “My Contraceptive Choice,” a tool tailored for women with hypertension, anxiety, and depression, was found to be easy to navigate by 91% of surveyed users, with 88% appreciating its educational content on contraceptive methods.^31^ This positive feedback was supported by a focus group, which emphasized the value of including detailed decision factors, such as weight gain, and a hybrid design for delivering personalized recommendations.^31^ A simulated evaluation of the decision aid reported 72% accuracy in addressing user preferences and needs.^31^

### 3.6 Reproductive health knowledge, pregnancy planning, and communication

Multiple studies highlighted the positive impact of online decision aids on reproductive health knowledge, pregnancy planning, and communication. The “MyFamilyPlan” web‐based preconception health education tool was associated with a significant increase in patient‐reported discussions of reproductive health with healthcare providers (odds ratio = 1.97, 95% confidence interval: 1.22–3.19).^36^ The “MyVoice:CF” tool, designed for women with cystic fibrosis, significantly improved reproductive health communication self‐efficacy, as indicated by an increase in the mean score on the Perceived Efficacy in Patient–Physician Interactions scale from 3.62 to 4.03 (*P* < 0.001).^35^ The proportion of participants discussing reproductive goals with their cystic fibrosis team increased from 33% to 56% after using “MyVoice:CF” (*P* = 0.046).^35^ Similarly, the “Roadmap to Parenthood,” designed for women with cancer, improved participants' confidence in discussing and managing fertility and family‐building issues, with participants indicating they felt encouraged to take a more active role in managing their fertility.^33^ “MyVoice:Rheum,” a tool for women with rheumatic diseases, provided salient, trustworthy, and clear information about family planning and rheumatic and musculoskeletal diseases.^34^ It was designed to promote patient autonomy, competence, and relatedness with their clinicians, and user feedback showed “MyVoice:Rheum” effectively achieved these goals.^34^ The “CHOICES” program, designed for individuals with sickle cell disease or trait, led to significant knowledge gains over 24 months compared to an e‐book control group (*P* = 0.004), highlighting the effectiveness of an interactive multimedia format for conveying complex health information.^14^

### 3.7 Contraceptive decision‐making, uptake, and behavior change

Some web‐based tools were specifically designed to influence contraceptive decision‐making, uptake, and behavior change. The “My Contraceptive Choice” decision aid, which takes into account the presence of chronic conditions such as hypertension, mental disorders, and coagulation disorders, was shown to be accurate, usable, and useful for selecting appropriate birth control methods.^31^ In an RCT, a “Computer‐Based Contraceptive Assessment Module” was found to influence contraceptive choice, with 75% of women receiving tailored materials and 78% of women receiving generic materials choosing an effective contraceptive method, compared to 65% in the control group.^37^ “Choosing Wisely” attracted an average of 24 users per day seeking information on birth control methods and their concerns.^38^, ^39^ While not directly focused on contraception, the “CHOICES” program for individuals with sickle cell disease or trait influenced reproductive behavior, with a higher proportion of participants in the “CHOICES” group choosing partners that reduced their risk of having children with sickle cell disease or trait, compared to the e‐book control group (*P* = 0.04).^14^

While many studies reported positive findings, there were mixed or inconclusive results reported regarding the impact on long‐term behavioral changes. For example, the “CHOICES” intervention did not significantly affect reproductive health intentions or behaviors over the 24‐month period.^14^ In addition, while “MyFamilyPlan” increased discussions with providers, it did not significantly affect behaviors such as initiating folate supplementation or changing birth control methods.^36^ A negative correlation was even observed between exposure to the “MyFamilyPlan” tool and initiating or changing birth control methods.^36^ Similarly, Healthy Beyond Pregnancy showed a trend towards increased compliance with postpartum visits, but this was not statistically significant.^32^

### 3.8 Risk of bias of included studies

The included studies showed a mixed range of methodological quality based on the MMAT assessment (Table 4). The mixed methods and qualitative studies generally demonstrated a lower risk of bias, meeting most of the relevant quality criteria. Conversely, the RCTs exhibited variability in quality, particularly concerning outcome data completeness and assessor blinding. Limitations were also observed in the quantitative descriptive studies, particularly regarding the representativeness of their samples. Overall, these patterns indicate moderate confidence in the evidence: while some studies provided methodologically sound findings, variability in design quality and sample representativeness limit the strength and generalizability of the findings.

**TABLE 4:** Risk of bias assessment of included studies using the Mixed Methods Appraisal Tool (MMAT).

Study type	Study	Mixed Methods Appraisal Tool (MMAT) criteria				
		Criterion 1	Criterion 2	Criterion 3	Criterion 4	Criterion 5
		Randomization appropriate?	Groups comparable at baseline?	Outcome data complete?	Assessors blinded?	Adherence to intervention adequate?
RCTs	Batra et al. (2018)^36^	Yes	Yes	Can't tell	No	Yes
	Gallo et al. (2016)^14^	Yes	Yes	Yes	Can't tell	Yes
	Garbers et al. (2012)^37^	Yes	Yes	Yes	No	Yes
	Himes et al. (2017)^32^	Yes	Yes	Yes	No	Yes
		Rationale for mixed methods adequate?	Components effectively integrated?	Outputs adequately interpreted?	Divergences adequately addressed?	Components adhere to quality criteria?
Mixed methods	Redman et al. (2023)^31^	Yes	Yes	Yes	No	Yes
	Benedict et al. (2022)^33^	Yes	Yes	Yes	No	Yes
		Sampling appropriate?	Sample representative?	Measurements appropriate?	Nonresponse bias low/addressed?	Analysis appropriate?
Quantitative descriptive	Kazmerski et al. (2023)^35^	Yes	Can't tell	Yes	Yes	Yes
	Nguyen et al. (2010)^38^	Yes	Can't tell	Yes	Can't tell	Yes
	Nguyen et al. (2011)^39^	Yes	Can't tell	Yes	Can't tell	Yes
		Approach appropriate?	Data collection adequate?	Findings derived from data	Interpretation substantiated?	Coherence in data process?
Qualitative	Talabi et al. (2022)^34^	Yes	Yes	Yes	Yes	Yes

Abbreviation: RCTs, randomized controlled trials.

## 4 DISCUSSION

This study reviewed nine distinct online decision aids designed to assist women with chronic conditions in making informed decisions about contraceptive and pregnancy planning options. Despite the limited number of studies identified and their varied focus across conditions like cancer, rheumatic diseases, cystic fibrosis, sickle cell disease, hypertension, mental health disorders, diabetes, and coagulation disorders, the findings highlight the significant potential of these online decision aids to improve informed contraceptive decision‐making, enhance reproductive health knowledge, support pregnancy planning, and strengthen patient–provider communication. While acknowledging some mixed results regarding long‐term behavioral changes, the findings suggest that these tools can still play an important role in facilitating informed and patient‐centered reproductive health choices.

A key strength of these decision aids lies in their personalized approach. By incorporating interactive elements like quizzes and questionnaires, tools such as “My Contraceptive Choice” and “Choosing Wisely”^31^, ^38^, ^39^ delivered tailored contraceptive recommendations, aligning with the growing emphasis on patient‐centered care. This customization acknowledges the impact of individual health profiles and preferences on contraceptive choices, moving beyond a one‐size‐fits‐all approach. Moreover, the integration of multimedia elements, as shown by the “CHOICES” program,^14^ offers diverse learning styles and has proven effective in communicating complex health information, particularly in the context of sickle cell disease. Decision aids like “Roadmap to Parenthood” and “MyVoice:Rheum”^33^, ^34^ further extended the scope of these aids by incorporating elements of values clarification and action planning, reflecting a more holistic understanding of reproductive decision‐making as a multifaceted process that extends beyond simple information acquisition.

Across the reviewed studies, a consistent theme that emerged was the high levels of feasibility, usability, and user satisfaction. For instance, “MyVoice:CF” demonstrated excellent usability, with high ratings for acceptability and appropriateness,^35^ while “Healthy Beyond Pregnancy” and “Roadmap to Parenthood” also received favorable usability scores, underscoring the importance of user‐centered design in digital health interventions.^32^, ^33^ These findings are consistent with the broader literature on digital health, which emphasizes that user‐friendly interfaces and engaging content are crucial for the adoption and sustained use of digital health tools.^40^, ^41^ However, it is important to acknowledge that high usability alone does not guarantee effectiveness in achieving desired health outcomes. Sustained behavior change additionally requires that interventions address determinants such as capability, opportunity, and motivation, as described in the COM‐B model.^42^ Integrating these elements can help ensure that decision aids move beyond usability to achieve lasting improvements in contraceptive behaviors.

Further, the varying levels of health literacy among women with chronic conditions represent a critical consideration in the design and implementation of these decision aids.^31^, ^37^ Health literacy can significantly affect an individual's ability to engage with, and benefit from, digital health interventions.^43^ While few decision aids, like the “Computer‐Based Contraceptive Assessment Module,” incorporated features such as audio‐computer‐assisted self‐interviewing to accommodate users with varying literacy levels,^37^ the effectiveness of these adaptations requires further investigation. Future studies should explore the relationship between health literacy and user engagement with online decision aids, as well as the development of strategies to tailor these tools to different literacy levels. This might involve using plain language, incorporating visual aids, or providing options for audio or video content. Addressing health literacy disparities is crucial to ensure that these promising tools are accessible and beneficial to all women, regardless of their educational background or ability to comprehend complex medical information.

The identified decision aids demonstrated considerable promise in improving reproductive health knowledge, pregnancy planning, and patient–provider communication. For instance, “MyFamilyPlan” was associated with a significant increase in patient‐reported discussions of reproductive health with providers,^36^ and “MyVoice:CF” improved participants' confidence in communicating their reproductive goals.^35^ These results suggest that online decision aids can empower women to become active participants in their reproductive health care, promoting shared decision‐making, a cornerstone of patient‐centered care.^44^ This empowerment is particularly crucial for women with chronic conditions, who often navigate complex medical landscapes and might face unique challenges in stating their reproductive needs and preferences.^45^ For example, the improved confidence reported by users of “Roadmap to Parenthood” and “MyVoice:Rheum” is particularly important, as women with cancer or rheumatic diseases might face fertility concerns and societal pressures that can complicate their reproductive decision‐making.^33^, ^34^

The findings on the impact of these decision aids on contraceptive decision‐making, uptake, and behavior change provided the most conjecture. While decision aids like “My Contraceptive Choice”^31^ and the “Computer‐Based Contraceptive Assessment Module”^37^ demonstrated effectiveness in influencing immediate contraceptive choices, aligning with previous study on the positive impact of decision aids in various healthcare contexts,^46^ the long‐term effects were less clear. Importantly, one study identified a negative correlation between decision aid use and contraception initiation.^36^ This warrants caution, as it suggests that in certain contexts, these tools might inadvertently reduce uptake rather than facilitate it. The mixed results from studies like “MyFamilyPlan”^36^ and “CHOICES,”^14^ which showed limited impact on sustained behavioral changes like folate supplementation or reproductive health intentions, further highlight the inherent challenges of behavior change. These findings suggest that while digital tools can be powerful channels for informed decision‐making, they might be insufficient on their own to ensure long‐term adherence to chosen methods or broader lifestyle modifications. Factors such as social support, access to healthcare services, and individual motivation likely play a significant role in sustaining behavioral changes.^42^

While this study provides valuable insights, it has certain limitations. The overall small number of studies eligible for review, the small sample sizes, and the heterogeneity of the target populations and decision aid features limit the generalizability of the findings. Additionally, we did not apply the GRADE approach to assess the quality of evidence, which further limits the strength of our conclusions. Future studies should focus on more robust sample sizes and more diverse representation to increase the external validity of the results. Further, the findings of many studies relied on self‐reported measures, which might introduce the potential for recall bias and social desirability bias. While self‐reports are valuable for understanding patient perspectives and intentions, they should be complemented by objective clinical and healthcare utilization outcomes, such as pharmacy records for contraceptive refills and clinical data on pregnancy rates, to provide a more balanced and reliable assessment. Combining both approaches would strengthen future evaluations by capturing both perceived and actual effects of decision aids.

## 5 CONCLUSION

This study highlighted the significant potential of online decision aids to transform contraceptive counseling and decision‐making for women with chronic conditions. These digital tools offer a personalized, engaging, and empowering approach to navigating complex reproductive choices, potentially improving patient–provider communication and fostering shared decision‐making. However, the mixed and negative results regarding long‐term behavioral change underscore the need for a more comprehensive approach. Such integration could include embedding decision aids into provider counseling, linking them with electronic medical records to generate personalized recommendations, and aligning them with structured follow‐up and broader health system supports to sustain behavior change. Future studies should prioritize optimizing the design of these decision aids, testing models of integration within healthcare workflows, and evaluating both behavioral outcomes and clinical endpoints across diverse populations.

## AUTHOR CONTRIBUTIONS

MLH conceptualized the study. BMG and MLH developed the search strategy. BMG and ND screened citations, extracted data, and carried out the quality assessment. BMG conducted data synthesis and drafted the manuscript. MLH reviewed and edited the manuscript for its contributions to the field. All authors have read and approved the final manuscript.

## FUNDING INFORMATION

We have not received any specific funding support to conduct this study.

## CONFLICT OF INTEREST STATEMENT

The authors have no competing interests to declare.

## Supporting information


**Table S1.** Search strategy.

Data S1.

## Data Availability

The data that support the findings of this study are available from the corresponding author upon reasonable request.
