# Supplementary material for: Online decision aids for contraceptive choices in women with chronic conditions: A systematic review
Source: Int J Gynaecol Obstet. 2025 Sep 27;172(3):1367–80. doi: 10.1002/ijgo.70555 (PMC12936640; doi:10.1002/ijgo.70555)
Supplement: Supplementary file 1 — Table S1. Search strategy. [file IJGO-172-1367-s002.docx]

**Supplemental Material**

**Supplementary Table 1: Search strategy**

| An extremely similar search strategy was followed on Medline (Ovid), Embase (Ovid) and Emcare (Ovid) and Maternity and Infant Care Database (Ovid) databases. | |
| --- | --- |
| **Search ID** | **Search Terms** |
|  | Decision Support Techniques/ |
|  | Decision Making/ |
|  | Decision Support Systems, Clinical/ |
|  | Counseling/ |
|  | Decision Making, Computer-Assisted/ |
|  | Choice Behavior/ |
|  | Health Education/ |
|  | Patient Education as Topic/ |
|  | Sexual counsel?ing.mp. |
|  | decision-mak*.mp. |
|  | decision aid*.mp. |
|  | decision tool*.mp. |
|  | educat*.mp. |
|  | informed choice.mp. |
|  | select*.mp. |
|  | shared decision*.mp. |
|  | tiered-effectiveness.mp. |
|  | 1 or 2 or 3 or 4 or 5 or 6 or 7 or 8 or 9 or 10 or 11 or 12 or 13 or 14 or 15 or 16 or 17 |
|  | Online Systems/ or Internet/ |
|  | Computer-Assisted Instruction/ |
|  | Technology/ |
|  | Telemedicine/ |
|  | (Online or web-based or website* or internet* or computer* or technology*).mp. |
|  | 19 or 20 or 21 or 22 or 23 |
|  | Contraception, Barrier/ or Contraception Behavior/ or Long-Acting Reversible Contraception/ or Contraception/ or Hormonal Contraception/ |
|  | Contraceptive Agents/ |
|  | Contraceptive Devices/ |
|  | Family Planning Services/ |
|  | Birth control*.mp. |
|  | Fertility regulation*.mp. |
|  | (Contracept* or Family plan* or Birth control*).mp. |
|  | 25 or 26 or 27 or 28 or 29 or 30 or 31 |
|  | 18 and 24 and 32 |
|  | limit 33 to english language |
| **CINAHL Complete (EBSCO)** | |
| [**Search ID#**](javascript:__doPostBack('ctl00$ctl00$MainContentArea$MainContentArea$historyControl$ReorderHistoryLink','')) | **Search Terms** |
| S1 | (MH "Decision Support Techniques") |
| S2 | (MH "Decision Making") |
| S3 | (MH "Decision Support Systems, Clinical") |
| S4 | (MH "Counseling") |
| S5 | (MH "Decision Making, Computer Assisted") |
| S6 | (MH "Decision Making, Shared") OR (MH "Decision Making, Patient") OR (MH "Decision Making, Family") |
| S7 | "Choice Behavior" |
| S8 | (MH "Health Education") |
| S9 | (MH "Patient Education") OR (MH "Patient Discharge Education") OR (MH "Patient Education (Iowa NIC)") |
| S10 | (MH "Sexual Counseling") OR (MH "Sexual Counseling (Iowa NIC)") OR (MH "Couples Counseling") |
| S11 | decision-mak* |
| S12 | "decision aid*" |
| S13 | decision tool* |
| S14 | educat* |
| S15 | "informed choice" |
| S16 | select* |
| S17 | "shared decision*" |
| S18 | tiered-effectiveness |
| S19 | (MH "Online Systems") |
| S20 | (MH "Internet") OR (MH "Internet Connections") OR (MH "Internet-Based Intervention") OR (MH "Website Development") OR (MH "World Wide Web Applications") OR (MH "World Wide Web") |
| S21 | (MH "Computer Assisted Instruction") |
| S22 | (MH "Technology") OR (MH "Educational Technology") OR (MH "Digital Technology") |
| S23 | (MH "Telemedicine") OR (MH "Telehealth") |
| S24 | Online or web-based or website* OR internet* OR computer* OR technology* |
| S25 | (MH "Contraception") OR (MH "Hormonal Contraception") OR (MH "Contraception Care (Saba CCC)") OR (MH "Family Planning: Contraception (Iowa NIC)") OR (MH "Contraceptives, Oral Combined") OR (MH "Contraceptive Agents") |
| S26 | (MH "Contraceptive Devices") OR (MH "Intrauterine Devices") |
| S27 | (MH "Family Planning") OR (MH "Family Planning Policy") OR (MH "Family Planning: Unplanned Pregnancy (Iowa NIC)") OR (MH "Family Planning: Infertility (Iowa NIC)") |
| S28 | ""Birth control*"" |
| S29 | "Fertility regulation*" |
| S30 | Contracept* OR "Family plan*" OR "Birth control*" |
| S31 | S1 OR S2 OR S3 OR S4 OR S5 OR S6 OR S7 OR S8 OR S9 OR S10 OR S11 OR S12 OR S13 OR S14 OR S15 OR S16 OR S17 OR S18 |
| S32 | S19 OR S20 OR S21 OR S22 OR S23 OR S24 |
| S33 | S25 OR S26 OR S27 OR S28 OR S29 OR S30 |
| S34 | S31 AND S32 AND S33 |
| S35 | S31 AND S32 AND S33 |
